# Supplementary material for: Effects of Irvingia gabonensis Extract on Metabolism, Antioxidants, Adipocytokines, Telomere Length, and Aerobic Capacity in Overweight/Obese Individuals
Source: Nutrients. 2022 Nov 3;14(21):4646. doi: 10.3390/nu14214646 (PMC9656030; doi:10.3390/nu14214646)
Supplement: Supplementary file 1 [file nutrients-14-04646-s001.zip › GMP_IG.pdf]

NSF INTERNATIONAL

789 N. Dixboro Road, Ann Arbor, Michigan 48105 USA
+1 800 673 6275

GMP Registered
Dietary Supplements

NSF International has assessed and confirmed compliance of

TCI Co., Ltd.

Facility: No. 12 Shennong Road, Changzhi Township, Pingtung County 90846, TWN

to NSF GMP Registration Program Requirements of NSF/ANSI 173, Section 8

which includes FSMA and cGMP (21 CFR 111), (21 CFR 117)

Print Date:	December 26, 2018
Certificate Number:	C0164873-173GMPMF-1
Initial Certification:	8/16/2013 1:01:38 PM
Expiration Date:	December 31, 2019

Cheryl Luther
General Manager, Dietary Supplements
and Beverage Quality

This certificate is the property of NSF International and must be returned upon request. For the most current and complete information, please access NSF's website (www.nsf.org).
