# Supplementary material for: Effects of Irvingia gabonensis Extract on Metabolism, Antioxidants, Adipocytokines, Telomere Length, and Aerobic Capacity in Overweight/Obese Individuals
Source: Nutrients. 2022 Nov 3;14(21):4646. doi: 10.3390/nu14214646 (PMC9656030; doi:10.3390/nu14214646)
Supplement: Supplementary file 1 [file nutrients-14-04646-s001.zip › Irvingia - SPEC (stamped).pdf]

Gateway Health Alliances, Inc.
"Partnerships for Nutritional Success"

4769 Mangels Blvd.
Fairfield, CA 94534
Phone: (707) 688-7999
Fax: (866) 569-9971
Email: shilkothari@hotmail.com

GHA IGOB131® Irvingia

IRV–300–P (80 Mesh Powder)

Specification Sheet

DESCRIPTION

Irvingia IRV–300 is a proprietary (US Patent 7,537,790) 10:1 aqueous extract of *Irvingia gabonensis* in a fine mesh powder for use as a dietary supplement in tableting, encapsulating and powder applications.

MANUFACTURE DATE

SPECIFICATIONS

Chemical Classification	Organic, Nutritive
Physical Classification	Powder, Non Fibrous
Genus, species	*Irvingia gabonensis*
Plant Part	Seeds
Color	Off-White
Odor	Slight Nutty
Taste	Nutty
Loss on Drying	Less than 5%
Solubility (water)	Partly Soluble
pH (1g/100ml water)	4.0 – 6.5
Active Ingredients	
Albumins (proteins) Method No. SIC / Q.M / 242	NLT 7%
Elagic Acid	0.96-1.44%
Solvents Used	Water
Heavy Metals:	
Pb (ppm)	Less than 0.5
As (ppm)	Less than 2
Cd (ppm)	Less than 0.2
Hg (ppm)	Less than 1
Particle Size	98% thru 80 Mesh
Bulk Density (g/cc)	NLT 0.3
Microbiological Assays:	
Total Plate Count (CFU/g)	Less than 5000
Yeast and Mold (CFU/g)	Less than 100
E. Coli (CFU/g)	Negative
Salmonella (CFU/g)	Negative
Staph. aureus (CFU/g)	Negative
Pesticide Assays:	
Organochlorine	Negative
Organophosphorous	Negative
Organonitrigen	Negative
N-Methyl Carbamates	Negative
Aflatoxins	Negative
Country of Origin	India
Country of Shipment	USA
Shelf Life	5 years when stored in tightly closed dark containers in a cool dry location. Limit exposure to air.
Expiration Date	

Sept 21, 2018

Gateway Health Alliances, Inc. Corporate Seal 6/22/05 California

PACKAGING

IRV–300–P is available in 25 kilogram quantities packaged in moisture, air and light-resistant containers.

Product Code IRV–300–P　　Research Code IGOB131　　Order Code IRV–300–P
